# PLAGL2 promotes the proliferation and migration of gastric cancer cells via USP37-mediated deubiquitination of Snail1

**DOI:** 10.7150/thno.47800

**Published:** 2021-01-01

**Authors:** Liang Wu, Ning Zhao, Zili Zhou, Jinhuang Chen, Shengbo Han, Xudan Zhang, Haijun Bao, Wenzheng Yuan, Xiaogang Shu

**Affiliations:** 1 Department of Gastrointestinal Surgery, Union Hospital, Tongji Medical College, Huazhong University of Science and Technology, Jiefang Road No. 1277, Wuhan 430022, China.; 2 Department of Emergency Surgery, Union Hospital, Tongji Medical College, Huazhong University of Science and Technology, Jiefang Road No. 1277, Wuhan 430022, China.; 3 Department of Gastrointestinal Surgery II, Renmin Hospital of Wuhan University, Zhang Zhidong Road No. 99 Wuhan 430060, China.

**Keywords:** PLAGL2, USP37, Snail1, GC, deubiquitination

## Abstract

**Rationale:** PLAGL2 (pleomorphic adenoma gene like-2), a zinc finger PLAG transcription factor, is aberrantly expressed in several malignant tumors. However, the biological roles of PLAGL2 and its underlying mechanism in gastric cancer (GC) remain unclear.

**Methods:** A series of experiments *in vitro* and *in vivo* were conducted to reveal the role of PLAGL2 in GC progression.

**Results:** The data revealed that PLAGL2 promotes GC cell proliferation, migration, invasion, and EMT *in vitro* and *in vivo*. Mechanistically, we demonstrated the critical role of PLAGL2 in the stabilization of snail family transcriptional repressor 1 (Snail1) and promoting Snail1-mediated proliferation and migration of GC cells. PLAGL2 activated the transcription of deubiquitinase USP37, which then interacted with and deubiquitinated Snail1 protein directly. In addition, GSK-3β-dependent phosphorylation of Snail1 protein is essential for USP37-mediated Snail1 deubiquitination regulation.

**Conclusions:** In general, PLAGL2 promotes the proliferation and migration of GC cells through USP37-mediated deubiquitination of Snail1 protein. This work provided potential therapeutic targets for GC treatment.

## Introduction

GC has been estimated to be the fifth most frequently diagnosed cancer and the third leading cause of cancer deaths worldwide in 2018, with about 1,000,000 new cases and 783,000 deaths 1. In particular, nearly more than half of the new cases occur in East Asia 1. Despite significant advances in the diagnosis and treatment of GC in the past decade, GC survival rates have not increased significantly. Metastasis, accounting for up to 90% of cancer-related deaths, is still the most incomprehensible part of cancer progression 2. However, little is known about the detailed molecular mechanisms involved in GC tumorigenesis and metastasis.

PLAGL2 is a highly similar homologue to PLAG1 structurally and functionally. The PLAG transcription factors specifically bind to the GRGGC(N)6-8RGGK consensus sequences in the target genes promoter to regulate its transcription 3. We previously clarified that PLAGL2 is implicated in the pathogenesis of Hirschsprung disease and colorectal cancer 4,5. PLAGL2 plays a carcinogenic role and has been reported in salivary gland tumors 6, colorectal cancer 7, and lung adenocarcinoma 8. PLAGL2 promotes the epithelial-mesenchymal transition (EMT) and mediates colorectal cancer metastasis by β-catenin-dependent regulation of ZEB1 5. PLAGL2 also enhances Wnt6 expression at the transcriptional level and promotes the progression of colorectal cancer 9. In addition, the PLAGL2-EGFR-HIF-1/2α signalling loop induces the malignant phenotype of liver cancer and the chemotherapy resistance of erlotinib 10. Although growing evidence has showed that PLAGL2 functions as a dominant oncogene in gastrointestinal cancers 7,11,12, the exact role of PLAGL2 and the underlying mechanism in GC remain largely unknown.

Metastasis is a major contributor to the mortality of cancer patients. Accumulating evidence indicates that the EMT initiates the metastatic progression of tumors 13. Therefore, a more comprehensive understanding of the EMT molecular mechanism will provide important insights to overcome cancer metastasis. It is well known that Snail1 plays a central role in inducing EMT. Snail1 triggers EMT by directly repressing E-cadherin expression and activating mesenchymal gene expression, such as fibronectin. Consistent with its function, Snail1 expression in human tumors is positively correlated with the tumor grade and enhanced invasiveness and metastasis 14,15. Therefore, it is critical to explore the exact molecular mechanisms, responsible for the aberrant expression of Snail1 in cancer.

Snail1 is a highly unstable protein that is subjected to the proteasome-ubiquitination degradation pathway. The E3-ubiquitin ligases transfer ubiquitin (Ub) from an E2 ubiquitin-conjugating enzyme to the substrate proteins with a lysine residue or a growing Ub chain. Multiple E3 ligases, including Skp-Culin-Fbox(SCF) E3 ligases 16,17, ubiquitin specific peptidase 27X(USP27X) 18, USP1 19, USP37 20,21, deubiquitinase3 (DUB3) 22 and A20 23, modulates Snail1 stability in various biological contexts. Snail1 proteasome-ubiquitination degradation, which is enhanced by β-TrCP1/FBXW1, requires phosphorylation of Snail1 by GSK3β 24. Although Snail1 functions have been well explored during tumor progression and metastasis, it remains unknown how Snail1 avoids ubiquitination and degradation in GC.

In this study, we demonstrated that PLAGL2 promoted the proliferation and migration of GC cells via USP37-mediated ubiquitination of Snail1 and revealed the precise regulatory mechanism of PLAGL2-mediated Snail1 stabilization. We clarified the enhanced PLAGL2 expression in GC tissues and elucidated the crucial effects of PLAGL2 on the proliferation, migration, and invasion of GC cells. Besides, we showed that PLAGL2 directly activates USP37 expression, which targets Snail1 to proteasome-ubiquitination degradation. More importantly, the identification of such a PLAGL2-USP37-Snail1 axis that regulates Snail1 ubiquitination and determination of the detailed mechanism will provide a potential novel therapeutic strategy for GC tumorigenesis and metastasis.

## Methods

### Patients and specimens

In total, 49 pairs of GC primary specimens were gathered from patients who had not been treated with radiotherapy or chemotherapy before the surgery. Two pathologists confirmed the diagnosis of GC in each case. This study was following the Declaration of Helsinki and approved by the Ethics Committee of Tongji Medical College, Huazhong University of Science and Technology.

### Cell culture and reagents

The GC cell lines SGC7901, BGC803, AGS, MKN45, BGC823, MGC803, HGC27 and the gastric mucosal epithelial cell lines GES-1 were obtained from American Type Culture Collection (ATCC, Manassas, VA, USA). SGC7901, AGS, BGC823, MKN45, BGC803, MGC803, HGC27, and GES-1 cells were cultured in RPMI-1640 medium (Gibco, Grand Island, NY, USA). All media were supplemented with 10% fetal bovine serum (FBS), 100 U/mL penicillin (Sigma-Aldrich, St Louis, MO, USA), and 100 mg/mL streptomycin (Sigma-Aldrich). Cycloheximide (CHX), proteasome inhibitor MG132, chloroquine diphosphate (CQ), and GSK-3β inhibitor CHIR-99021 were bought from Selleck Chemicals (Houston, TX, USA). Alkaline phosphatase (CIP) was obtained from Roche (Roche Applied Science, Indianapolis, IN, USA). The GSK-3β inhibitor LiCl was obtained from Sigma Aldrich.

### Western blotting (WB) and immunoprecipitation (IP) analysis

WB and IP analyses were performed as described previously 25. Detailed antibody information is provided in Table S1.

### *In vivo* ubiquitination assay

The indicated plasmids or siRNAs were transiently transfected into HEK293T or GC cells. The ubiquitination assay was performed as previously described 25.

### Quantitative real-time polymerase chain reaction (qRT-PCR)

Total RNA from cells or specimens was extracted using TRIzol reagent (TaKaRa, Kyoto, Japan). According to the manufacturer's instructions, the qRT-PCR assay was performed using the SYBR Green PCR kit (TaKaRa). The primer sequences are given in Table S2.

### Cell proliferation assay

Cell proliferation assays were performed with CCK-8 reagents (Dojondo Laboratories, Kumamoto, Japan) according to the manufacturer's instructions. A total of 2 × 10^3^ cells were plated into 96-well plates. Then, the cells were incubated with CCK8 for 2 h at scheduled time points.

### Colony formation assay

GC cells were plated in 6-well plates and cultured in the corresponding medium for 2 weeks. The culture medium was changed every 3 days. Cell colonies were stained with 1% crystal violet and then counted.

### Cell cycle analysis

For cell cycle analysis, cells were harvested and washed with cold PBS, and fixed with 75% cold ethanol. Before analysis with a BD FACS flow cytometer, the cells were incubated with propidium iodide (PI) (Invitrogen, Gaithersburg, MD, USA) for 30 min.

### Cell migration and invasion assays

Wound-healing and Transwell assays were used to measure the migration and invasion of cells. These assays were performed as previously described 26.

### Immunofluorescence (IF) assay

The IF assay was carried out, as described previously 25. Primary antibodies specific for USP37 (1:100) (Proteintech, Rosemont, IL, USA) and Snail1 (1:100) (Santa Cruz Biotechnology, Dallas, Texas, USA) were applied. Fluorescence images were acquired.

### Immunohistochemistry (IHC)

IHC analysis was conducted as described elsewhere 27. According to the staining area scores and staining intensity, the quantification of IHC staining results was evaluated as previously described 27.

### Transfection

Human Lenti-shPLAGL2-GFP (PLAGL2 shRNA 5′-GACCCATGATCCTAACAAA-3′, Genechem, Shanghai, China), Lenti-PLAGL2-Flag (OBiO Technology, Shanghai, China) and their controls were utilized in our study. To establish stable cell lines, 5 × 104 cells were seeded in 6-well plates. After lentivirus infection, single-cell clonal isolates were maintained in a culture medium with 5 μg/mL puromycin (Sigma-Aldrich) for 2 weeks. The transfection efficiency was determined by WB and qRT-PCR analyses. USP38, DUSP18, His-USP37, His-USP37-C350S, Flag-Ubiquitin, MYC-Ubiquitin, HA-Snail1, USP37 promoter (wild-type, mut1, mut2 and mut3), different deletion mutants of HA-Snail1, wild-type Snail1 (Snail1-WT) and Snail1 mutant Snail1-6SA were cloned into the pcDNA3 vector. Short interfering RNAs (siRNAs) for Snail1 (siSnail1), USP37 (siUSP37), GSK-3β(siGSK-3β) and their controls were synthesized by RiboBio (Guangzhou, China). The targeted sequences were as follows: siSnail1, 5′-CCTAGTCAGCCACCTTTAA-3′, siUSP37#1, 5′-CAGACACTATGGAAACTGA-3′, siUSP37#2, 5′-AAGCGTGGTTTACTTACAA-3′ and siGSK-3β, 5′-GGAGTGCACA TATGGCAAT-3′. Transfections of plasmids and siRNAs were performed using Lipofectamine 3000 (Invitrogen). Effective transfection was confirmed by WB.

### Chromatin immunoprecipitation (ChIP)

The ChIP assay was carried out using the ChIP kit (Cell Signaling Technology, Danvers, MA, USA). All experimental procedures were performed following the kit instructions. The ChIP DNA was amplified by qRT-PCR; the products of qRT-PCR were electrophoresed on a 2% agarose gel. The primers for the USP37 promoter region sequence were as follows: forward primer (5'-GAAACTGTTGGTCAGCGCAA-3'), reverse primer (5'-CTCCTAATTCCCGGTGTCGG-3').

### Measurement of promoter-reporter activity

GC cells and HEK293T cells were transfected with the designated USP37 promoter reporters combined with the PLAGL2 siRNA or PLAGL2 plasmid. The cotransfection of the β-actin/Renilla luciferase reporter gene was used to normalize USP37 promoter activity. According to the manufacturer's instructions, the luciferase activity was quantified using a dual luciferase assay system (Promega, Madison, WI, USA).

### Xenograft assay

The mouse experiments were approved by the Institutional Animal Care and Use Committee of Tongji Medical College, Huazhong University of Science and Technology. BALB/c male nude mice (5 weeks old) were purchased from Beijing Vital River (Beijing, China). For the tumorigenesis assay, 6×10^6^ cells in 200 μL of PBS, were implanted subcutaneously into the groin of the mice (6 mice for each group). Tumor size was calculated with the following formula: V= 0.5× (length) × (width)^2^. All mice were sacrificed 4 weeks after inoculation. For metastasis assay, 4×10^6^ cells suspended in 150 μL of PBS, were injected into the tail veins of the mice (7 mice for each group), and all mice were killed after 6 weeks. The metastatic nodules were counted by two observers and then confirmed by hematoxylin-eosin (HE) staining.

### Statistical analysis

All *in vitro* experiments were performed three times or more. The results are presented as the mean ± SD. The statistical analyses were completed by GraphPad Prism 8 or SPSS 20.0 software. The data analysis was conducted using Student's t-test for comparisons between groups. The χ^2^ test was utilized to evaluate the association between the target genes expression and clinicopathological features. Pearson's correlation test assessed correlation analysis. Differences were considered significant at * *p*<0.05, ** *p*<0.01, and *** *p*<0.001. n.s: no significance.

## Results

### PLAGL2 is highly expressed in GC

**Figure 1:**
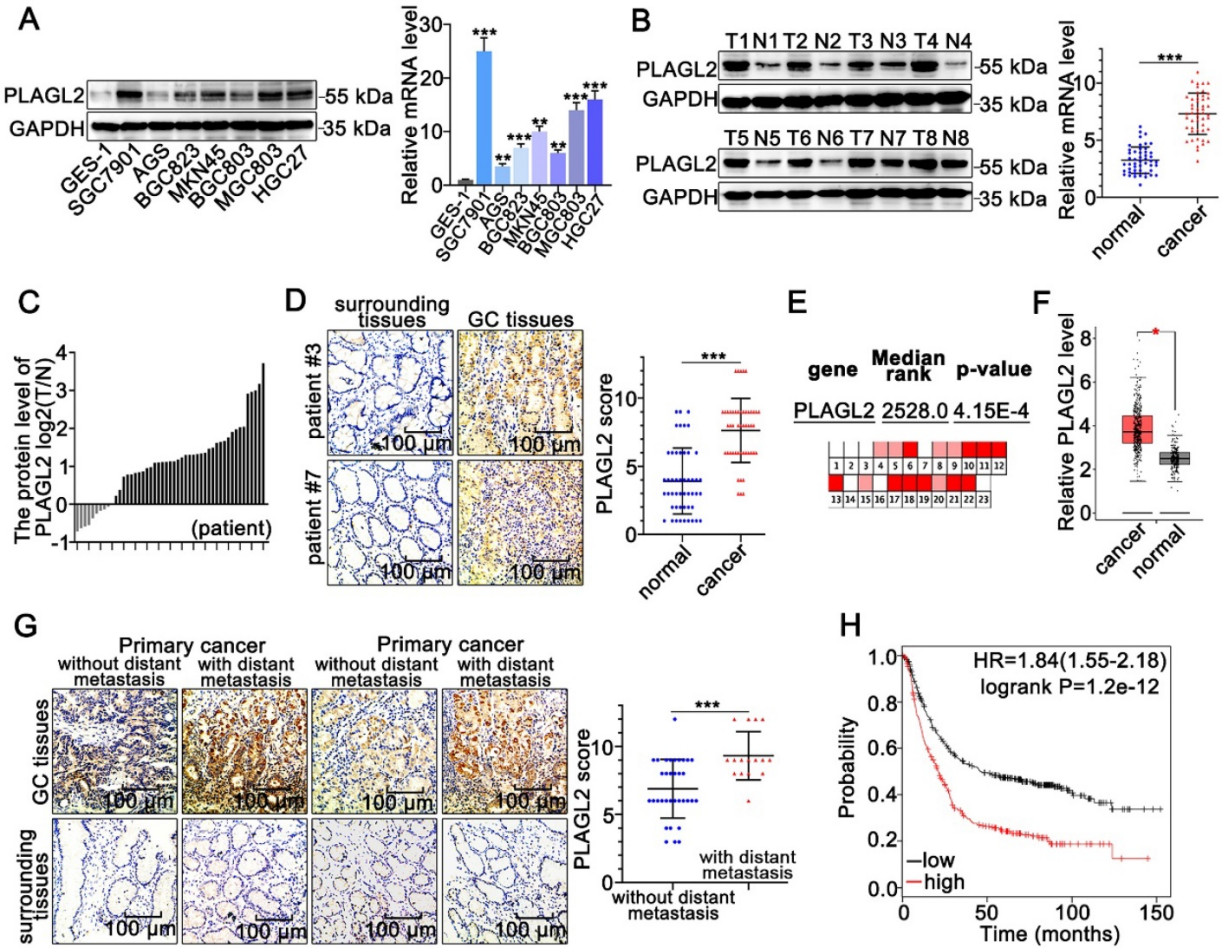
** PLAGL2 is highly expressed in GC.** (**A**) WB and qRT-PCR analyses of PLAGL2 expression in human GC cell lines and GES-1. (**B**) WB and qRT-PCR analyses of PLAGL2 expression levels in clinical GC specimens. T, GC tissue; N, paired normal tissues. (**C**) Quantitative analysis of relative PLAGL2 expression in 49 paired GC tissues. (**D**)Representative IHC images of the PLAGL2 expression levels in paired GC tissues. Scale bars, 100 µm. (**E**) A meta-analysis of the PLAGL2 gene expression derived from the Oncomine database. (**F**) A box plot reflecting the PLAGL2 expression in GC specimens and normal specimens was obtained from the GEPIA database (**G**) Representative IHC images of the PLAGL2 expression levels in primary GC tumors without metastasis vs. GC tumors with metastasis. Scale bars, 100 µm. (**H**) Kaplan-Meier survival analysis of overall survival based on PLAGL2 expression in GC patients.

To clarify the role of PLAGL2 in GC tumorigenesis, we first examined the PLAGL2 expression profiles in clinical GC specimens and human GC cell lines. PLAGL2 expression in most GC cell lines was markedly higher than its expression in gastric mucosal epithelial cell line GES-1(Figure 1A). Significantly, higher levels of PLAGL2 mRNA and protein were observed in primary GC tumors than in matched paraneoplastic tissues (Figure 1B-D), which is consistent with the data obtained from the Oncomine database 28 (Figure 1E) and GEPIA database 29 (Figure 1F). IHC analysis further showed that PLAGL2 was highly expressed in most GC samples with distant metastasis than those without distant metastasis (Figure 1G). Associations between PLAGL2 expression and clinicopathological parameters are shown in Table 1. Notably, high PLAGL2 expression was significantly linked to tumor invasion depth (*P* = 0.033) and lymph node metastasis (*P* = 0.003). Data from the Kaplan-Meier Plotter database 30 showed that patients with high PLAGL2 levels displayed poor overall survival (Figure 1H). Overall, these results suggest that PLAGL2 is highly expressed in GC patients and is related to poor prognosis.

### PLAGL2 promotes the proliferation, migration, and invasion of GC cells

**Figure 2:**
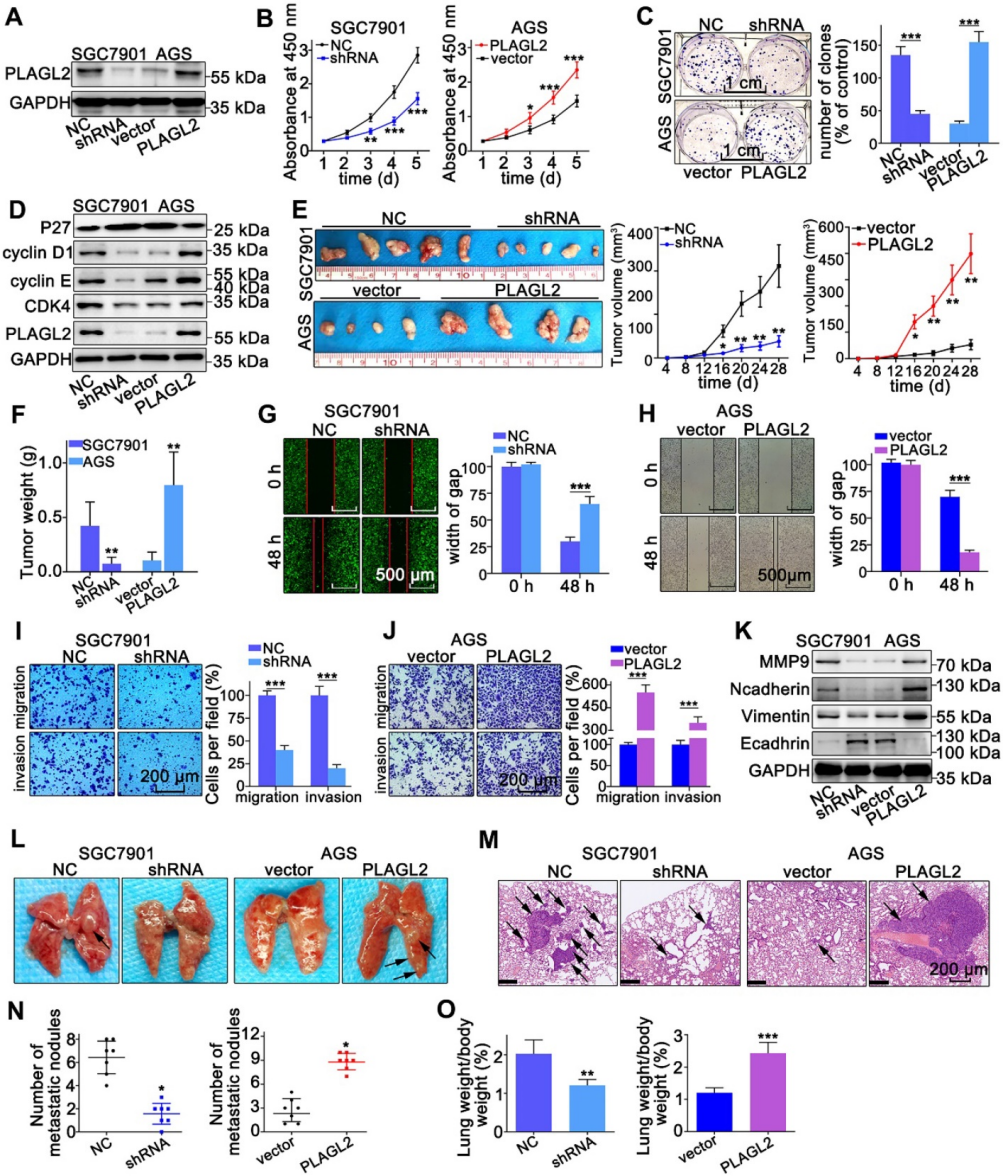
** PLAGL2 promotes the proliferation, migration, and invasion of GC cells.** (**A**) WB analysis of PLAGL2 expression in SGC7901 transfected with Lenti-shPLAGL2 and AGS transfected with Lenti-PLAGL2. (**B-C**) The cell proliferation was examined by CCK8 (**B**) and colony formation assays (**C**) in SGC7901 and AGS cells. Scale bars, 1cm. (**D**) WB analysis of the expression level of crucial cell cycle regulatory proteins. (**E**) Representative images of the corresponding xenograft. The growth curve of the tumors in the different groups. (**F**) The weight of tumors in different groups. (**G-H**) The cell migration was examined by the wound-healing assay. Pictures were taken at 0 and 48 h, respectively. Scale bars, 500μm. (**I-J**) The migration and invasion capacities of GC cells were also evaluated with Transwell assays. Scale bars, 200 µm. (**K**) WB analysis of the expression level of EMT-related proteins. (**L**) Representative images of visible lung metastases. The metastatic nodules were indicated with arrows. (**M**) Representative images of the corresponding HE staining. Scale bars, 200 µm. (**N**) Numbers of metastatic nodules. (**O**) The ratio of lung weight/body weight.

To explore the oncogenic role of PLAGL2 in GC, we established stable PLAGL2 knockdown (SGC7901-shRNA) and overexpression (AGS-PLAGL2) cells. Transfection efficiency was confirmed by WB and qRT-PCR (Figure 2A and Figure S1A). The CCK-8 assay demonstrated that silencing PLAGL2 significantly inhibited cell proliferation, whereas enforced PLAGL2 expression enhanced the growth of GC cells (Figure 2B). Additionally, colony formation assays indicated that silencing of PLAGL2 expression resulted in much smaller and fewer colonies, whereas higher PLAGL2 expression promoted clonogenicity (Figure 2C). The cell cycle analysis results showed that PLAGL2 decreased the G0G1 fraction and increased the S and G2M fractions (Figure S1B). Additionally, stable PLAGL2 depletion decreased the expression levels of vital cell proliferation regulatory proteins (cyclinD1, cyclin E, and CDK4), and enhanced p27kip1 (a key cell cycle inhibitor) expression (Figure 2D and Figure S1C). To extend the *in vitro* results, we explored the effect of PLAGL2 on tumor growth using a xenograft model. The volume and weight of the shPLAGL2 group were significantly reduced compared to the control. The volume and weight of tumors formed by cells with higher PLAGL2 expression were dramatically increased than the matched group (Figure 2E-F). Furthermore, the expression levels of cell cycle regulatory proteins and p27kip1 in tumors formed by cells with stable PLAGL2 depletion are consistent with the results of WB in PLAGL2 knockdown cells (Figure S1D-E). In general, these results indicate that PLAGL2 is a regulator of proliferation in GC.

Next, we explored the metastatic potential of GC cells with different PLAGL2 expression levels. As shown in Figure 2G-J, PLAGL2 enhanced the migration and invasion of GC cells *in vitro*. Given that matrix metalloproteinase (MMP) family proteins and EMT-related proteins participate in tumor cell migration and invasion, we examined the transcript and protein level of MMP9, N-cadherin, Vimentin, and E-cadherin in cells with modified PLAGL2 expression levels. As shown in Figure 2K and Figure S1C, SGC7901-shRNA cells displayed a higher expression level of E-cadherin and a lower level of N-cadherin, Vimentin, and MMP9 than SGC7901-NC cells. In contrast, PLAGL2 overexpression in AGS cells triggered the expression of MMP9, N-cadherin and Vimentin, accompanied by a concomitant decrease in E-cadherin. Furthermore, the EMT-related proteins were investigated by IHC analysis (Figure S1D-E), which was conducted with mouse tumors formed by the corresponding cells. An experimental lung metastasis assay was performed to assess the *in vivo* role of PLAGL2 in tumor metastasis (Figure 2L-M). The lung weight and number of metastatic nodules increased significantly in tumor xenografts that overexpress PLAGL2 (Figure 2N-O). Together, these findings indicate the crucial role of PLAGL2 in GC metastasis and invasion.

### PLAGL2 stabilizes Snail1 protein by inhibiting its ubiquitination

**Figure 3:**
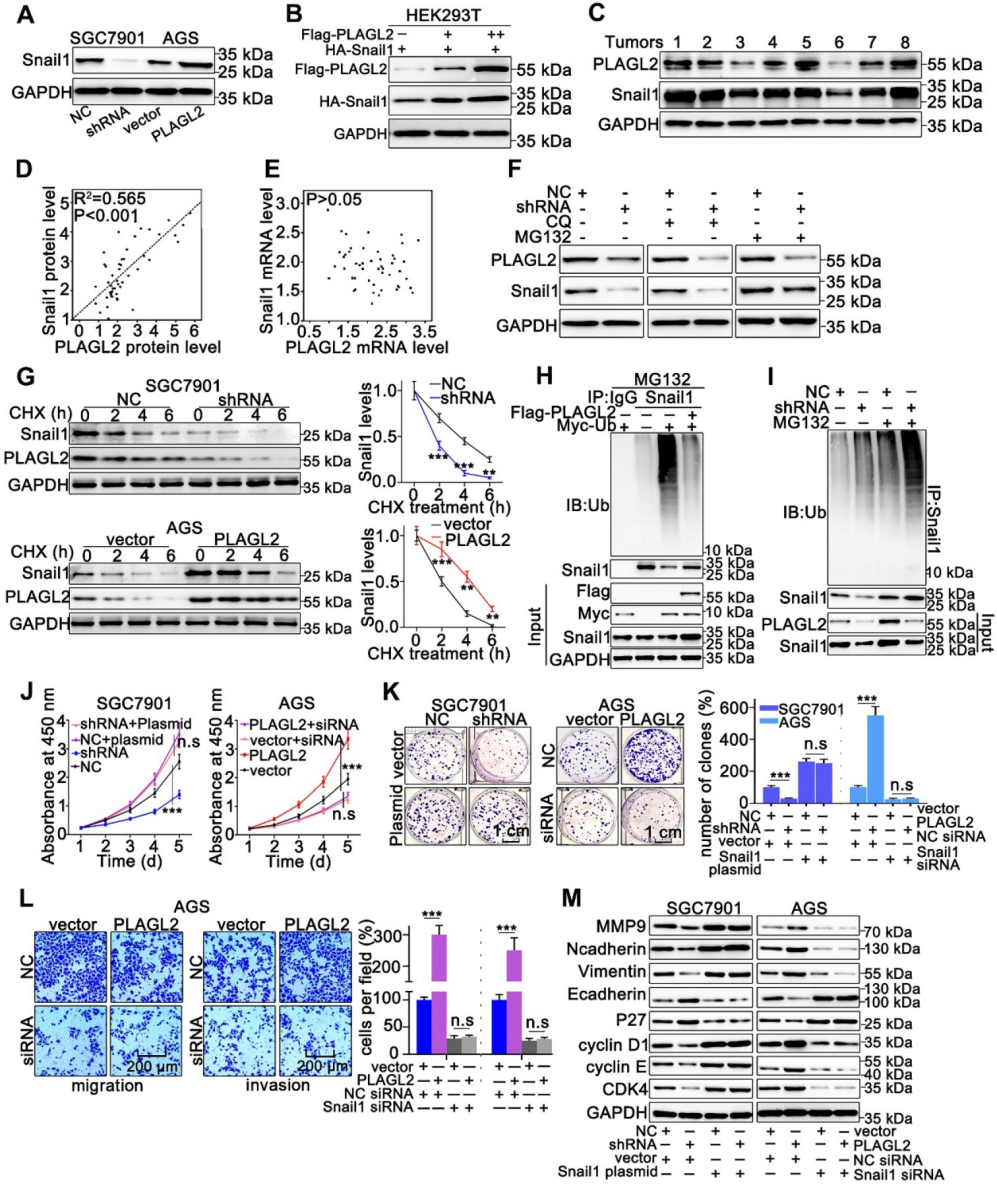
** PLAGL2 stabilizes Snail1 protein by inhibiting its ubiquitination.** (**A**) WB analysis of Snail1 expression of in PLAGL2 knockdown and overexpression cells. (**B**) WB analysis of exogenous Snail1 in HEK293T cells co-expressing PLAGL2 and Snail1. (**C**) WB analysis of protein levels of PLAGL2 and Snail1 in clinical GC specimens. (**D**) The correlation between PLAGL2 protein and Snail1 protein in GC tissues. (**E**) The correlation between PLAGL2 mRNA and Snail1 mRNA in GC tissues. (**F**) WB analysis of Snail1 level in PLAGL2 knockdown cells treated with MG132 and CQ. (**G**) CHX chase analysis of Snail1 protein half-life in PLAGL2 knockdown SGC7901 cell and PLAGL2 overexpression AGS cell. (**H-I**) Ubiquitination assays of endogenous Snail1 in the lysates from AGS cells transfected with Flag-PLAGL2 (**H**) or AGC7901 cells stably expressing PLAGL2 shRNA (**I**). (**J-K**) The stable PLAGL2 knockdown (SGC7901-shRNA) cell was transfected with Snail1 plasmid, and stable overexpression (AGS-PLAGL2) cell was transfected with the Snail1 siRNA. The role of Snail1 in PLAGL2-induced proliferation was examined by CCK8 (**J**) and colony formation assays (**K**). (**L**) Transwell assays detected the effect of Snail1 on PLAGL2-induced migration. Scale bars, 200 µm (l). (**M**) WB analysis of the expression level of EMT-related proteins and critical cell cycle regulatory proteins in cotransfected SGC7901 and AGS cells.

Snail1 plays a particularly crucial role in the proliferation and migration of GC cells, thus, we examined whether PLAGL2 exerts oncogenic effects on the proliferation and migration of GC cells in a Snail1-dependent manner. To examine the possible role of PLAGL2 in regulating Snail1 expression, we detected Snail1 protein levels in cells with different PLAGL2 expression levels. The depletion of PLAGL2 significantly inhibited Snail1 protein expression in SGC7901 cells, and enhanced PLAGL2 expression, which considerably elevated Snail1 protein levels in the AGS cells (Figure 3A). Notably, no statistically significant changes in the Snail1 mRNA levels were observed (Figure S2A), suggesting that PLAGL2 modulates Snail1 expression post-transcriptionally. Similarly, ectopic PLAGL2 expression in HEK293T cells dramatically elevated exogenous Snail1 protein levels in a dose-dependent manner (Figure 3B). Besides, PLAGL2 expression levels were positively correlated with Snail1 protein levels in GC samples (Figure 3C and Figure S2B). We observed a linear correlation between PLAGL2 and Snail1 protein levels in clinical GC specimens, and this linear correlation was not observed between PLAGL2 and Snail1 mRNA levels (Figure 3D-E). We speculate that PLAGL2 might regulate the degradation pathway of Snail1 protein, and that the autophagolysosome pathway and ubiquitin-proteasome pathway are the main pathways for protein degradation. We treated GC cells with ubiquitin-proteasome pathway inhibitor MG132 and autophagolysosome pathway inhibitor chloroquine diphosphate (CQ), and found that MG132 could reverse PLAGL2 regulation of Snail1 protein to a certain extent (Figure 3F). To examine whether PLAGL2 regulates the Snail1 protein stability, we detected the abundance of Snail1 in GC cells treated with CHX. Overexpression of PLAGL2 in the AGS cells significantly increased the stability and half-life of Snail1. Accordingly, the silencing of PLAGL2 in SGC7901 cells significantly destabilized the Snail1 protein (Figure 3G). We further explored the role of PLAGL2 in Snail1 ubiquitination. In the presence or absence of MG132, enforced PLAGL2 expression inhibited Snail1 ubiquitination in the AGS cells (Figure S2C), whereas silencing PLAGL2 promoted Snail1 ubiquitination (Figure 3H-I). The above data together show that PLAGL2 stabilizes the Snail1 protein by inhibiting its ubiquitination.

To further explore whether PLAGL2 induces the proliferation, migration, and invasion of GC cells in a Snail1-dependent manner, we utilized either the specific Snail1 siRNA or plasmid to conduct studies in SGC7901 and AGS cells. The effect of Snail1 siRNA and plasmid on the proliferation, migration, invasion, and EMT markers was also examined. Snail1 siRNAs impaired cell proliferation in both AGS-vector and AGS-PLAGL2 cells (Figure 3J-K and Figure S2D). Conversely, the Snail1 plasmid promoted cell proliferation in both SGC7901-NC and SGC7901-shRNA cells (Figure 3J-K). A Transwell assay was then conducted to explore the role of Snail1 siRNA in GC cell motility. The results showed that PLAGL2 overexpression increased GC cell motility, whereas Snail1 siRNA blocked this function (Figure 3L and Figure S2E). Moreover, the regulation of proliferation and metastasis-related genes caused by PLAGL2 depletion was impaired by the Snail1 plasmid, whereas the regulation resulting from PLAGL2 overexpression could be opposed by Snail1 siRNA (Figure 3M and Figure S2F-G).

### USP37 interacts with and deubiquitinates Snail1 directly

**Figure 4:**
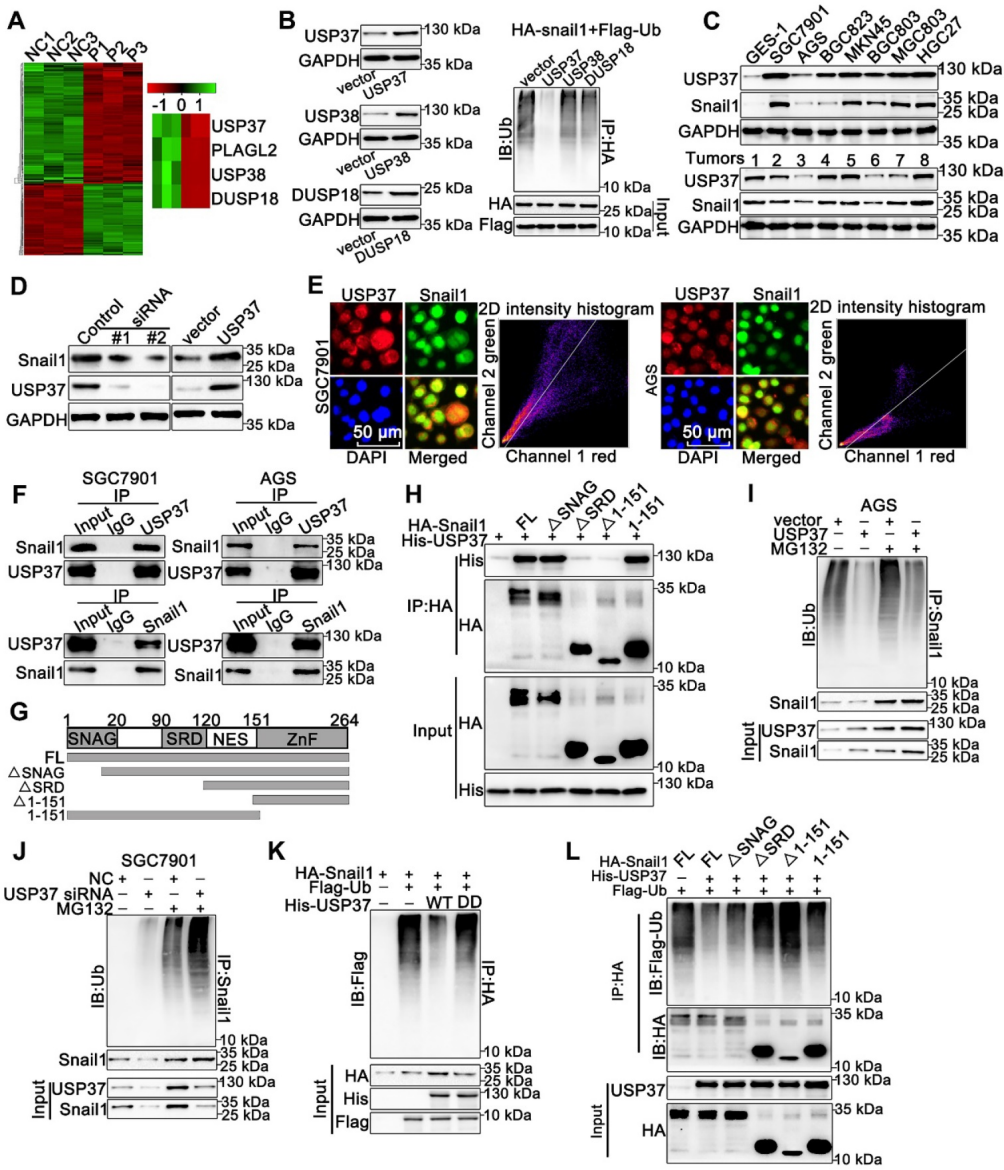
** USP37 interacts with and deubiquitinates Snail1 directly.** (**A**) Microarray analysis for mRNAs was performed with RNA extracted from SGC7901 NC and SGC7901 shRNA cells. (**B**) Ubiquitination assays of endogenous Snail1 in HEK293T cell, which was cotransfected with HA-Snail1, Flag-Ubi, and one of the three DUBs (USP37, USP38, and DUSP18). (**C**) WB analysis of protein levels of USP37 and Snail1 in clinical GC specimens and human GC cell lines. (**D**) WB analysis of protein levels of Snail1 and USP37 in SGC7901 cells transfected with two independent USP37 siRNAs and AGS cells expressing USP37 plasmid. (**E**) The representative images of IF staining of Snail1 (green) and USP37 (red) were shown in SGC7901 and AGS cells. Scale bars, 50 µm. (**F**) Reciprocal Co-IP and WB assays indicated the interaction between endogenous USP37 and Snail1 in AGS and SGC7901 cells. (**G**) Schematic diagram of the Snail1 full-length and deletion mutant plasmid. (**H**) HEK293T cells were cotransfected with USP37-His and the full length or truncation mutants of HA-Snail1. Cell lysates were immunoprecipitated with anti-HA antibody, and its production was analyzed by WB analysis with anti-His antibody (**I-J**) Ubiquitination assays of endogenous Snail1 in the lysates from AGS cells transfected with the USP37 plasmid (**I**) or AGC7901 cells transfected with the USP37 siRNA (**J**). (**K**) Ubiquitination assays of endogenous Snail1 in the lysates from HEK293T cells, which were cotransfected with the His-USP37-WT or His-USP37-DD, HA-Snail1 and Flag-Ub expressing plasmids. (**L**) Ubiquitination assays of endogenous Snail1 in the lysates from HEK293T cells, which were cotransfected with USP37-His and the full length or truncation mutants of HA-Snail1.

PLAGL2 mainly serves as a transcription factor and unlikely acts as a deubiquitinase to directly mediate Snail1 ubiquitination. Therefore, we speculate that some DUBs might be responsible for Snail1 ubiquitination induced by PLAGL2. The mRNA microarray analysis was conducted using total RNA isolated from SGC7901 NC and SGC7901 shRNA cells (n=3), and we screened three DUBs from the differentially expressed genes (Figure 4A). We then investigated the influence of the DUBs on Snail1 ubiquitination. Among these three DUBs, only USP37 significantly diminished the ubiquitination of Snail1, suggesting that USP37 may be a potential deubiquitinase of Snail1 (Figure 4B). USP37 protein expression was positively correlated with Snail1 protein levels in GC cell lines and GC tissues (Figure 4C). We then examined the effect of USP37 on Snail protein expression. As shown in Figure 4D and Figure S3A, depletion of USP37 in SGC7901 and HEK293T cells significantly reduced Snail1 protein expression, whereas enhanced USP37 expression in AGS and HEK293T cells dramatically upregulated the Snail1 protein levels. As expected, USP37 did not affect the mRNA levels of Snail1 (Figure S3B). We further investigated the interaction between USP37 and Snail1. USP37 and Snail1 proteins are mainly colocalized in the nucleus (Figure 4E). Also, the reciprocal co-immunoprecipitation analysis demonstrated that endogenous USP37 and Snail1 bound to each other in SGC7901 and AGS cells (Figure 4F). To map the binding protein domains for Snail1 interacting with USP37, we constructed a series of HA-Snail1 truncations and found that the serine-rich domain (SRD) within Snail1 is required for its interaction with USP37 (Figure 4G-H). To examine whether USP37 regulates Snail1 protein stability, we detected the abundance of Snail1 in GC cells treated with CHX. USP37 considerably increased the stability and half-life of Snail1 in the AGS cells. Accordingly, the silencing of USP37 in SGC7901 cells significantly destabilized the Snail1 protein (Figure S3C-D). We also assessed the impact of USP37 on Snail1 ubiquitination. Overexpression of USP37 significantly reduced the endogenous ubiquitination of Snail1 in the AGS cells. Accordingly, silencing endogenous USP37 promoted Snail1 ubiquitination in the presence or absence of MG132 (Figure 4I-J). Moreover, overexpression of USP37-C350S, a DUB dead mutant USP37 (USP37DD) 31, did not alter Snail1 ubiquitination (Figure 4K). Given that the SRD domain is essential for the interaction between USP37 and Snail1, we evaluated its effect on Snail1 ubiquitination in HEK293T cells. As expected, ΔSRD and Δ1-151 could not be deubiquitinated by USP37 (Figure 4L). Therefore, the SRD domain is also crucial for USP37-mediated Snail1 deubiquitination and the interaction between USP37 and Snail1. Overall, these results demonstrate that USP37 interacts with and deubiquitinates Snail1 directly.

### USP37 targets Snail1 for degradation in a GSK-3β phosphorylation-dependent manner

**Figure 5:**
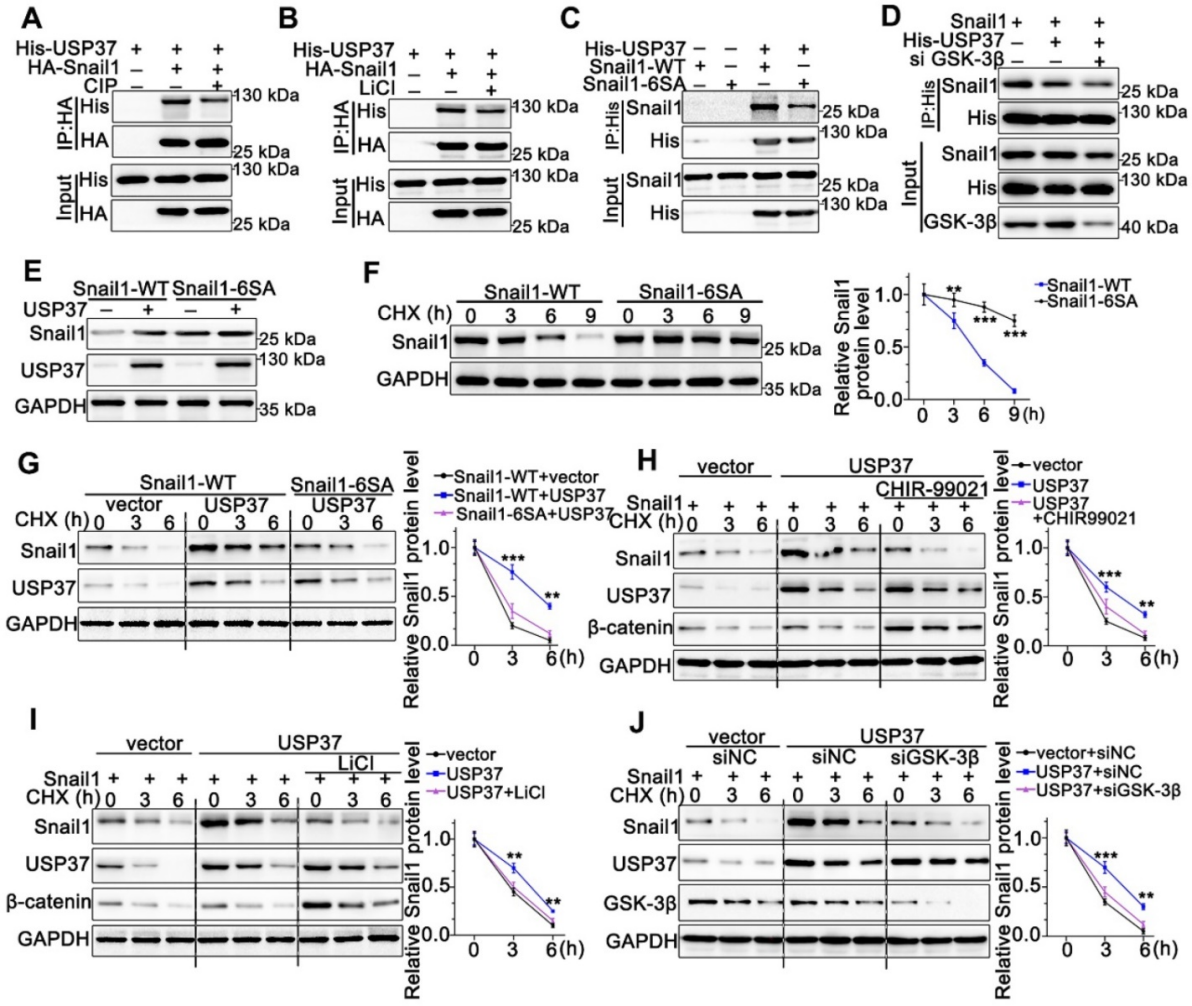
** USP37 targets Snail1 for degradation in a GSK-3β phosphorylation-dependent manner.** (**A**) Co-IP and western blot assays showed the mutual binding ability between USP37 and Snail1 in HEK293T cells treated either with or without CIP. (**B**) Co-IP and western blot assays showed the mutual binding ability between USP37 and Snail1 in HEK293T cells treated either with or without LiCl (**C**) Co-IP and western blot assays showed the mutual binding ability between USP37 and Snail1 in HEK293T cells cotransfected with His-USP37 and Wild-type Snail1 or Snail1-6SA mutant. (**D**) Co-IP and western blot assays showed the mutual binding ability between USP37 and Snail1 in HEK293T cells cotransfected either with or without si GSK3β (**E**) The WB analysis reflecting the expression regulation of USP37 on Wild-type Snail1 and Snail1-6SA mutants in HEK293T cells. (**F**) Pulse-chase assays of wild-type Snail1 or Snail1-6SA mutant in HEK293T cells. (**G**) Pulse-chase assays of Snail1 in HEK293T cells cotransfected with USP37 and Wild-type Snail1 or Snail1-6SA mutant. (**H**) Pulse-chase assays of Snail1 in HEK293T cells, which were transfected with His-USP37 plasmid and were treated either with or without LiCl. **(I)** Pulse-chase assays of Snail1 in HEK293T cells, which were transfected with His-USP37 plasmid and were treated either with or without CHIR-99021. (**J**) Pulse-chase assays of Snail1 in AGS cells, which were cotransfected with His-USP37 plasmid and siGSK-3β.

As we know, the proper phosphorylation of target proteins by specific kinases is critical for DUBs to identify their substrates for subsequent ubiquitination and degradation 32. To explore whether the interaction between Snail1 and USP37 requires Snail1 phosphorylation, we utilized CIP to remove phosphate groups from the substrate proteins. Strikingly, Co-IP experiments showed that the interaction between USP37 and Snail1 was strongly impeded by CIP intervention (Figure 5A). Since GSK-3β is the major kinase involved in Snail1 phosphorylation in the SRD region, the GSK-3β inhibitor LiCl was used to assess GSK-3β inhibition in the interaction of USP37 and Snail1. As shown in Figure 5B, LiCl treatment also significantly disrupted the USP37 and Snail1 interaction. Thus, we constructed the Snail1 mutant Snail1-6SA, and its six putative GSK-3β phosphorylation sites within the SRD domain were mutated from Ser to Ala 33. Snail1-6SA demonstrated a significantly decreased interaction with USP37 as expected (Figure 5C). The specific GSK-3β siRNAs were further used to investigate the role of GSK-3β in the interaction between Snail1 and the USP37 protein. Co-IP results showed that GSK-3β siRNAs diminished approximately 60% of the interaction of USP37 and Snail1 (Figure 5D). Furthermore, we confirmed the role of GSK-3β in the USP37-mediated stabilization of Snail1. As shown in Figure 5E, USP37 remarkably elevated the protein level of wild-type Snail1 but had no significant impact on Snail1-6SA. Notably, the Snail1-6SA exhibited higher stabilization than wild-type Snail1 with CHX treatment (Figure 5F). However, Snail1-6SA was strongly resistant to USP37-mediated Snail1stabilization (Figure 5G). Intervention with GSK-3β inhibitors CHIR-99021 or LiCl also displayed a strong resistance to USP37-mediated stabilization of Snail1 (Figure 5H-I). Besides, silencing of endogenous GSK-3β impeded USP37-mediated stabilization of Snail1 in AGS cells (Figure 5J). These results consistently support that GSK-3β-dependent phosphorylation of Snail1 is vital for its USP37-mediated stabilization.

### PLAGL2 modulates Snail1 stability by activating USP37 transcription

**Figure 6:**
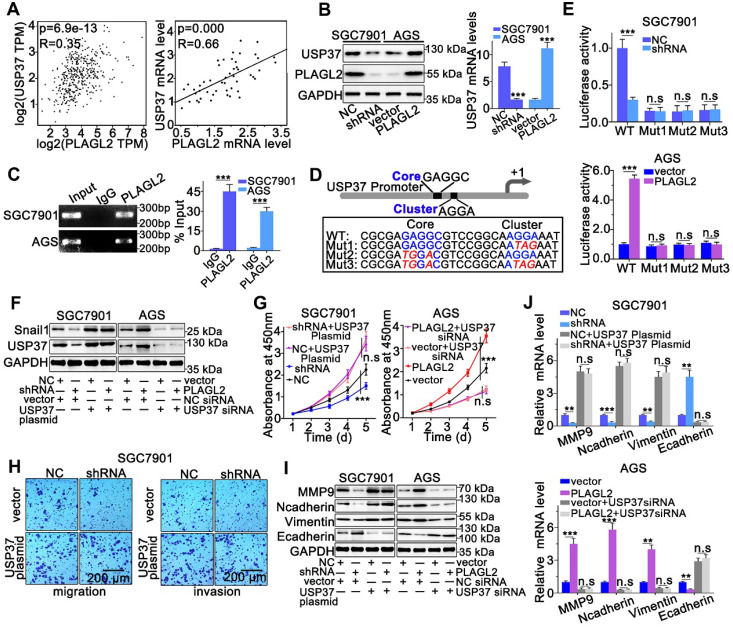
** PLAGL2 modulates Snail1 stability by activating USP37 transcription.** (**A**) A significant positive correlation between PLAGL2 mRNA and USP37 mRNA in GC tissues could be observed both in GEPIA database(left) and our data(right). (**B**) WB and qRT-PCR analyses of Snail1expression in PLAGL2 knockdown SGC7901 cell and PLAGL2 overexpression AGS cell. (**C**) The ChIP DNA was amplified by qRT-PCR, and then the products of qRT-PCR were electrophoresed on a 2% agarose gel. (**D**) The schematic representation of USP37 promoter. The sequences of wild-type and mutant PLAGL2 binding sites were indicated. (**E**) Luciferase activity assays were performed in PLAGL2 knockdown SGC7901 cell and PLAGL2 overexpression AGS cell, which were then transfected with wild type (USP37-WT) or mutant-type (USP37-mut) USP37 promoter-reporter plasmids. (**F**) WB of Snail1 protein level in SGC7901 cell cotransfected with Lenti-shPLAGL2 and USP37 plasmid and AGS cell cotransfected with Lenti-PLAGL2 and USP37 siRNA. (**G-H**) The stable PLAGL2 knockdown (SGC7901-shRNA) cell was transfected with USP37 plasmid, and stable overexpression (AGS-PLAGL2) cell was transfected with the USP37 siRNA. The role of USP37 in PLAGL2-induced proliferation was examined by CCK8 (**G**). Transwell assays detected the effect of USP37 on PLAGL2-induced migration. Scale bars, 200 µm (**H**). (**I**) WB analysis of the expression level of EMT-related proteins in cotransfected SGC7901 and AGS cells. (**J**) The qRT-PCR analysis of the expression level of EMT-related genes in cotransfected SGC7901 and AGS cells.

We sought to identify whether PLAGL2 serves as a transcription factor, directly regulating the transcriptional level of USP37 in GC. First, we investigated the PLAGL2, and USP37 mRNA levels in GC by utilizing the GEPIA database. We observed a linear correlation between PLAGL2 and USP37 mRNA levels. The USP37 mRNA levels were also linearly correlated with PLAGL2 mRNA levels in clinical GC specimens (Figure 6A). Moreover, silencing PLAGL2 decreased the mRNA level of USP37 in SGC7901 cells, whereas PLAGL2 overexpression increased the USP37 mRNA level in AGS cells (Figure 6B). This finding was further confirmed at the protein level in cells with different PLAGL2 expression levels (Figure 6B). Given that PLAGL2 can specifically bind to the GRGGC(N)6-8RGGK consensus sequences in the promoter, one potential PLAGL2 transcriptional binding site was identified in the USP37 promoter region. The ChIP assay revealed that PLAGL2 was bound to the USP37 promoter region (Figure 6C). To further determine the transcriptional regulation of USP37 by PLAGL2, we constructed promoter-reporter plasmids, that harbored wild-type and mutations of the PLAGL2 binding site (Figure 6D). These promoter-reporter plasmids were transfected into cells with different PLAGL2 expression levels, and the luciferase activity was subsequently detected. Results indicated that PLAGL2 can significantly increase wild-type promoter activity but demonstrated no effect on mutant promoters (Figure 6E), and the core and G-cluster were found to be critical for PLAGL2 binding.

**Figure 7:**
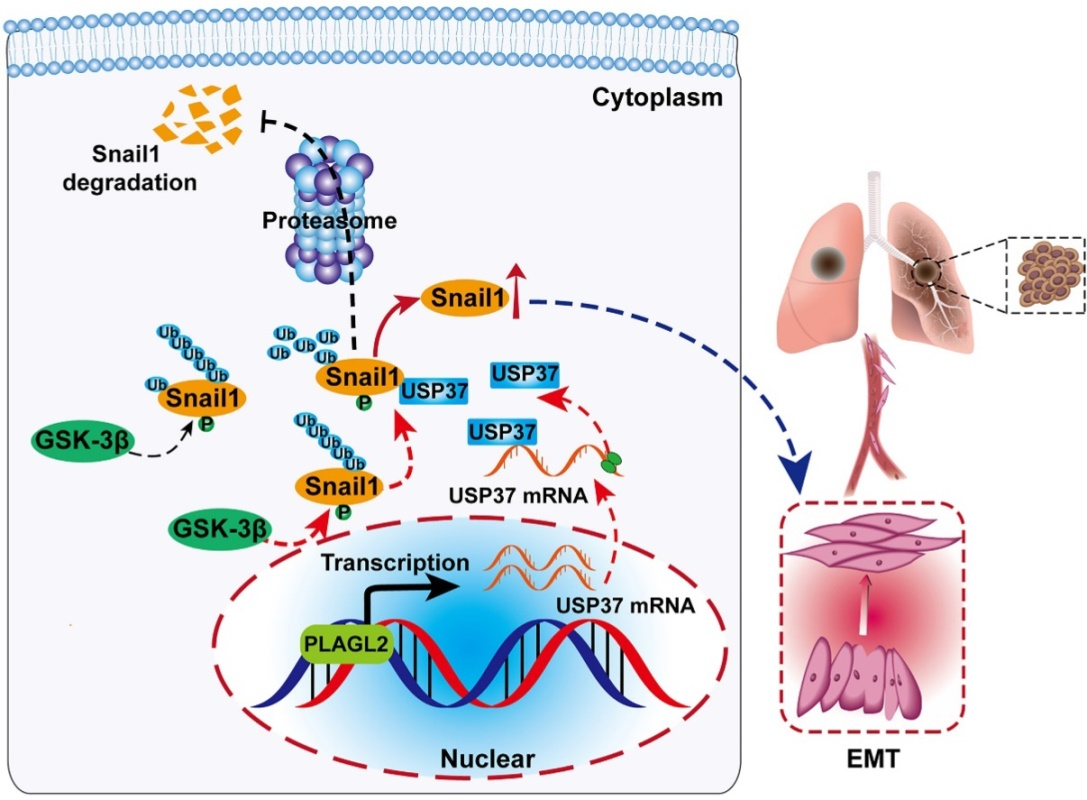
** Schematic diagram of the molecular mechanism of PLAGL2 promoting proliferation and migration of GC cells.** Schematic diagram of the molecular mechanism of PLAGL2 promoting proliferation and migration of GC cells. High expression of PLAGL2 transcriptionally activates USP37 expression to protect Snail1 from degradation by the proteasome.

We further clarified whether PLAGL2 regulated Snail1 protein expression in a USP37-dependent manner, and we utilized either the specific USP37 siRNA or plasmid to conduct studies in SGC7901 and AGS cells. After cotransfection with the USP37-plasmid, the expression level of Snail1 protein was significantly restored, and the expression level of Snail1 protein between SGC7901-NC and SGC7901-shRNA did not show statistically significant differences (Figure 6F). The expression of Snail1 mRNA in cotransfected cells was detected by qRT-PCR. The results showed that compared with the corresponding control group, there was no significant difference in the level of Snail1 mRNA among the above groups (Figure S4A). To further explore whether PLAGL2 regulated the expression of Snail1 protein in a USP37-dependent manner, it mediated the proliferation, migration, and invasion of GC cells. CCK8 experiments and Transwell migration and invasion experiments were performed on the cells after cotransfection. After cotransfection with USP37-plasmid, the cell proliferation, migration, and invasion abilities were significantly restored, and after cotransfection with USP37-siRNAs, the cell proliferation, migration, and invasion abilities were significantly inhibited (Figure 6G-H and Figure S4B-D). We found that after cotransfection of USP37-plasmid, there was no significant difference in the expression levels of cell cycle regulatory genes and EMT-related genes between SGC7901-NC and SGC7901-shRNA (Figure 6J-I and Figure S4E-F). After cotransfection of USP37-siRNA, there was no significant difference in the expression levels of cell cycle regulatory genes and EMT-related genes between AGS-vector and AGS-PLAGL2 (Figure 6J-I and Figure S4E-F). These results collectively suggest that PLAGL2 transcriptionally activates USP37 expression to increase Snail1 protein levels, which in turn mediates the proliferation, migration, and invasion of GC cells (Figure 7A).

## Discussion

PLAGL2 mainly acts as a transcription factor that has been implicated in the progression of multiple types of tumors. In our study, we observed that PLAGL2 is upregulated in GC cells and GC specimens. Subsequent *in vitro* and *in vivo* experiments showed that overexpression of PLAGL2 significantly promotes cancer cell proliferation and metastasis, indicating the carcinogenic role of PLAGL2 in regulating GC progression. Consistent with our results, recent studies have revealed that PLAGL2 serves as an oncogene to induce lung cancer8, colorectal cancer 5,7,12, and breast cancer 34. Of note, we found that the underlying mechanism by which PLAGL2 functions as a promoter for GC proliferation and migration is by stabilizing Snail1. We also determined that USP37 interacts with and deubiquitinates Snail1 directly, which is transcriptionally activated by PLAGL2.

As an essential transcription factor, Snail1 plays a crucial role in proliferation, metastasis, tumor recurrence, and metabolism 14. Snail1 is strictly controlled at the transcriptional, translational, and posttranslational levels 35. The Snail1 protein levels change rapidly in response to cellular stress, mostly dependent on protein stability regulation 16. Enhanced Snail1 expression leads to several cancers, including GC. Herein, we determined that PLAGL2 is a crucial and novel regulator that upregulates the Snail1 protein by inhibiting the ubiquitin-proteasome pathway, and this is one of our key findings. PLAGL2 overexpression significantly increased Snail1 protein levels to promote GC cell proliferation and metastasis. Although PLAGL2 does not function as a deubiquitinase to impede Snail1 ubiquitination, PLAGL2 transcriptionally activates USP37, which finally interacts with and deubiquitinates Snail1 directly.

The deubiquitinase USP37 is involved in the tumorigenesis process, specifically cell cycle progression 36-38, EMT 39, chemosensitivity 39 and cell self-renewal 39. USP37 was initially identified as an effective modulator of G1/S transition 36,38. Previous studies reported that USP37 increased the Warburg effect and cell viability by repressing ubiquitin-mediated degradation of c-Myc 31. Moreover, overexpression of USP37 in breast cancer promoted stemness, cell invasion, and chemoresistance by deubiquitinating the glioma-associated oncogene 1 protein 39. In the past year, two other deubiquitinating enzymes, USP27X and USP1, have been reported to impede ubiquitin-mediated degradation of Snail1 in various biological contexts 18,19. USP27X modulates tumor chemoresistance and invasion through deubiquitination and stabilization of Snail1 18. USP1 is a Snail1 deubiquitinase and increases metastatic ability and chemoresistance 19. In our study, Snail1 was identified as a new substrate of USP37 in GC, which is an important finding. Overexpression of USP37 in SGC7901-shRNA cells restores Snail1 protein levels, which shows that the USP37-Snail1 axis plays a vital role in PLAGL2-mediated Snail1 stabilization.

Generally, proper phosphorylation of substrates is vital for the DUB-mediated proteasomal degradation pathway. In terms of the overall intracellular environment, Snail1 6SA protein is more stable than Snail1-WT protein. However, in cells with high expression of USP37, phosphorylated Snail1 promotes the mutual binding of USP37 and Snail1, and the ability of USP37 to bind to phosphorylated Snail1 is significantly enhanced. USP37 exerts its powerful deubiquitinating enzyme function, and the stability of Snail1-WT Significantly higher than Snail1 6SA. It further shows that USP37 regulation of Snail1 protein stability requires the participation of GSK-3β-mediated phosphorylation. Moreover, USP37 exerts a strong deubiquitination and stabilization effect on the phosphorylated Snail1 protein. These results further revealed that the SRD domain of Snail1 is responsible for USP37-mediated Snail1 deubiquitination and GSK-3β-induced phosphorylation and could be a potent therapeutic target for GC. In this study, we demonstrated that SRD is required for its interaction with USP37. In addition, Snail1 phosphorylated by GSK-3β, is easier to combine with USP37. Phosphorylated Snail1, combined with USP37 is more stable. However, the Snail1 mutant, Snail1-6SA, is much more stable than wild type Snail1. Liu *et al.* found that Snail1 phosphorylated by GSK-3β, interacts with SPSB3, showing lower stability 33. Perhaps, the phosphorylation of Snail1 mainly promotes its binding to E3 ligases. Phosphorylated Snail1 binds to deubiquitinating enzymes, which will increase its stability, whereas binding to the ubiquitin enzyme will accelerate its degradation.

In summary, our study reveals a PLAGL2-USP37-Snail1 axis representing a critical mechanism in the proliferation and migration of GC cells (Figure 7A). Hence, blocking this axis may be a potent therapeutic strategy against GC.

## Supplementary Material

Supplementary figures and tables.

Supplementary material-The mRNA microarray analysis.

## Figures and Tables

**Table 1:** Clinicopathological analysis of PLAGL2 expression in GC

Parameters	n	PLAGL2		
		+	-	*P*-value
**Age (years)**				
<60	31	26	5	0.149
≥60	18	11	7	
**Gender**				
Male	30	24	6	0.564
Female	19	13	6	
**Size of tumor**				
<3 cm	20	12	8	0.079
≥3 cm	29	25	4	
**Differentiation**				
Well-moderate	23	15	8	0.214
Poor	26	22	4	
**T Stages**				
T1-T2	18	10	8	0.033*
T3-T4	31	27	4	
**Metastasis**				
**N Stages**				
N0	14	6	8	0.003*
N1-3	35	31	4	
**M Stages**				
M0	34	24	10	0.398
M1	15	13	2	

## Abbreviations

PLAGL2: Pleomorphic adenoma gene like-2
USP37: ubiquitin-specific peptidase 37
qRT-PCR: Real-time quantitative polymerase chain reaction
IF: Immunofluorescence
ChIP: Chromatin immunoprecipitation
Snail1: snail family transcriptional repressor 1
GC: gastric cancer
EMT: epithelial-mesenchymal transition
HE: Hematoxylin and eosin
CHX: cycloheximide
CQ: chloroquine diphosphate
