# Supplementary material for: PLAGL2 promotes the proliferation and migration of gastric cancer cells via USP37-mediated deubiquitination of Snail1
Source: Theranostics. 2021 Jan 1;11(2):700–14. doi: 10.7150/thno.47800 (PMC7738862; doi:10.7150/thno.47800)
Supplement: Supplementary file 1 — Supplementary figures and tables. [file thnov11p0700s1.pdf]

Table S1. Primary antibodies for WB

Antibody	Concentration	Specificity	Company
PLAGL2	1:1000	Rabbit polyclonal	Abcam
E-cadherin	1:1000	Mouse monoclonal	Proteintech
Vimentin	1:1000	Mouse monoclonal	Proteintech
N-cadherin	1:1000	Rabbit monoclonal	Proteintech
P27kip1	1:1000	Mouse monoclonal	Cell Signaling Technology
Cyclin-E	1:1000	Rabbit monoclonal	Cell Signaling Technology
His	1:2000	Mouse monoclonal	Proteintech
GAPDH	1:2000	Rabbit monoclonal	Proteintech
Flag	1:2000	Mouse monoclonal	Proteintech
HA	1:2000	Mouse monoclonal	Proteintech
CDK4	1:1000	Rabbit monoclonal	Proteintech
Cyclin-D1	1:1000	Mouse monoclonal	Proteintech
Snail1	1:1000	Rabbit polyclonal	Proteintech
USP37	1:1000	Rabbit polyclonal	Proteintech
MYC	1:1000	Mouse monoclonal	Proteintech
GSK-3β	1:1000	Rabbit monoclonal	Cell Signaling Technology
Ub	1:1000	Rabbit Polyclonal	Proteintech
USP38	1:1000	Rabbit Polyclonal	Proteintech
DUSP18	1:1000	Mouse Polyclonal	Abcom
MMP9	1:1000	Rabbit Polyclonal	Proteintech

Table S2. Sequence of primers for Quantitative reverse transcription-PCR

Gene	Forward primer (5’------3’)	Reverse primer (5’------3’)
PLAGL2	GAGTCAAGTGAAGTGCCAATGT	TGAGGGCAGCTATATGGTCTC
E-cadherin	CGAGAGCTACACGTTCACGG	GGGTGTCGAGGGAAAAATAGG
Vimentin	CGAAACTTCTCAGCATCACG	GCAGAAAGGCACTTGAAAGC
N-cadherin	TCAGGCGTCTGTAGAGGCTT	ATGCACATCCTTCGATAAGACTG
P27kip1	AACGTGCGAGTGTCTAACGG	CCCTCTAGGGGTTTGTGATTCT
Cyclin-D1	GCTGCGAAGTGGAAACCATC	CCTCCTTCTGCACACATTTGAA
Cyclin-E	AAGGAGCGGGACACCATGA	ACGGTCACGTTTGCCTTCC
CDK4	TCAGCACAGTTCGTGAGGTG	GTCCATCAGCCGGACAACAT
Cyclin-D1	GTGCTGCGAAGTGGAAACC	ATCCAGGTGGCGACGATCT
MMP9	AGACCTGGGCAGATTCCAAAC	CGGCAAGTCTTCCGAGTAGT
GAPDH	AGAAGGCTGGGGCTCATTTG	AGGGGCCATCCACAGTCTTC

Supplementary Figure Legends

Figure S1. PLAGL2 promotes the proliferation, migration, and invasion of GC cells in vitro and in vivo. (**A**) The qRT-PCR analysis of PLAGL2 expression in SGC7901 transfected with Lenti-shPLAGL2 and AGS transfected with Lenti-PLAGL2. (**B**) The cell cycle results of PLAGL2 knockdown SGC7901 cell and PLAGL2 overexpression AGS cell. (**C**)The qRT-PCR analysis of the expression level of crucial cell cycle regulatory proteins and EMT-related proteins in PLAGL2 knockdown SGC7901 cell and PLAGL2 overexpression AGS cell. (**D**) Representative IHC images of the expression of essential cell cycle regulatory proteins and EMT-related proteins in the corresponding xenograft. Scale bars, 50μm. (**E**) The IHC scores of the expression of essential cell cycle regulatory proteins and EMT-related proteins in the corresponding xenograft.

Figure S2. PLAGL2 stabilizes Snail1 protein by inhibiting its ubiquitination. (**A**) The qRT-PCR analysis of the Snail1 expression level in PLAGL2 knockdown SGC7901 cell and PLAGL2 overexpression AGS cell. (**B**) WB analysis of protein levels of PLAGL2 and Snail1 in clinical GC specimens. (**C**) Ubiquitination assays of endogenous Snail1 in the lysates from PLAGL2 overexpression AGS cell. (**D**) WB analysis of Snail1 expression in SGC7901 transfected with two independent Snail1 siRNAs and AGS transfected with Snail1 plasmid. (**E**)Transwell assays detected the effect of Snail1 on PLAGL2-induced migration. Scale bars, 200μm. (**F-G**) The qRT-PCR analysis of the expression level of EMT-related genes and critical cell cycle regulatory genes in cotransfected SGC7901 and AGS cells.

Figure S3. USP37 interacts with and deubiquitinates Snail1 directly. (**A**) WB analysis of protein

levels of Snail1 and USP37 in HEK-293T cell transfected with two independent USP37 siRNAs and USP37 plasmid. (**B**) The qRT-PCR analysis of mRNA levels of Snail1 in HEK-293T cell transfected with USP37 siRNAs and USP37 plasmid, in SGC7901 cell transfected with two independent USP37 siRNAs and in AGS cell expressing USP37 plasmid. (**C-D**) The stability of Snail1 by USP37 was estimated using the pulse-chase assay.

Figure S4. PLAGL2 modulates Snail1 stability by activating USP37 transcription. (**A**) The qRT-PCR analysis of Snail1 mRNA level in SGC7901 cell cotransfected with Lenti-shPLAGL2 and USP37 plasmid and AGS cell cotransfected with Lenti-PLAGL2 and USP37 siRNA. (**B-D**) Transwell assays detected the effect of USP37 on PLAGL2-induced migration. Scale bars, 200μm. The average number of cells per field was calculated. (**E**) WB analysis of crucial cell cycle regulatory proteins' expression level in cotransfected SGC7901 and AGS cells. (**F**) The qRT–PCR analysis of the expression level of key cell cycle regulatory genes in cotransfected SGC7901 and AGS cells.

Figure S1

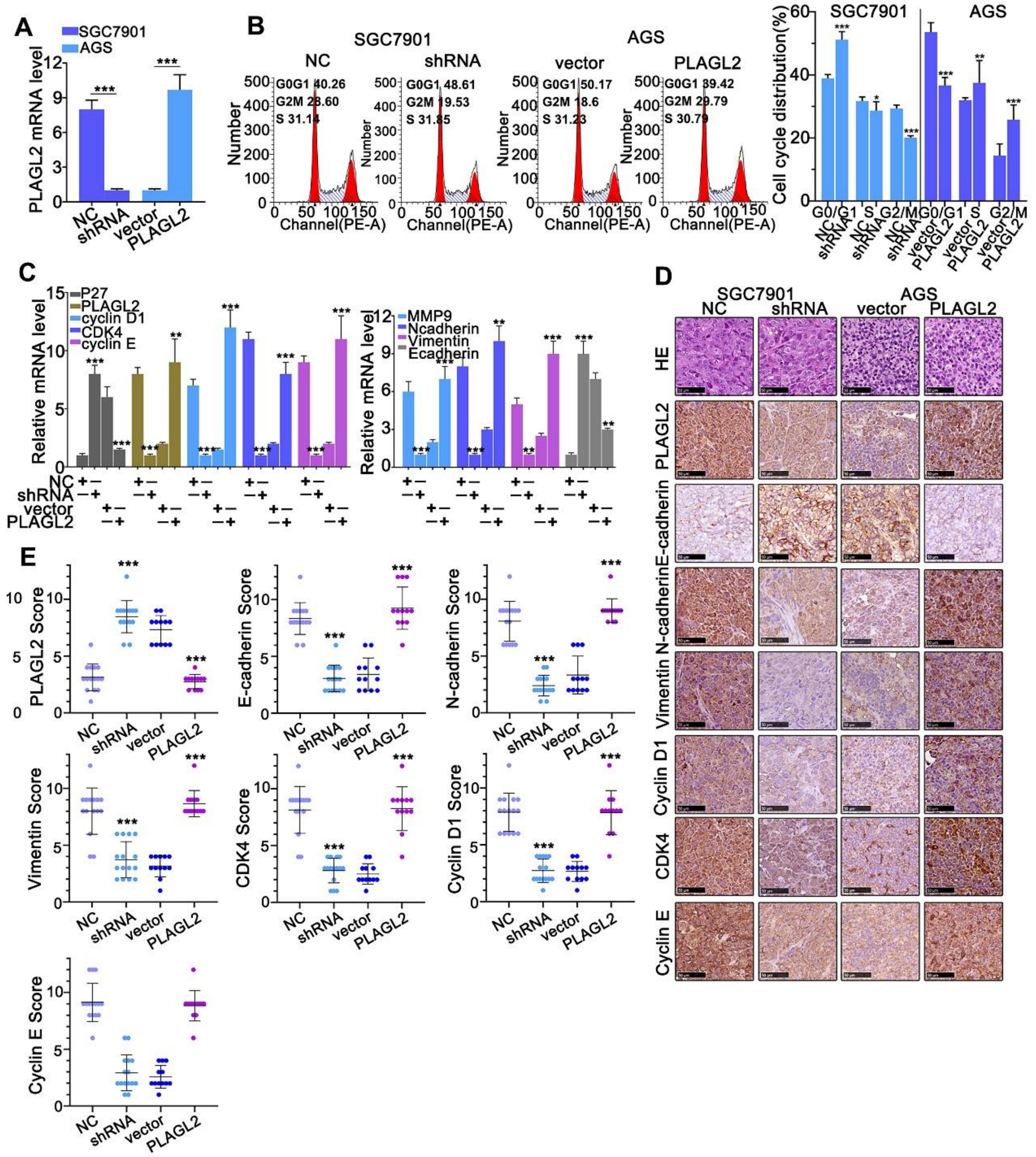
A
SGC7901
AGS
PLAGL2 mRNA level
NC
shRNA
vector
PLAGL2
B
SGC7901
AGS
NC
shRNA
vector
PLAGL2
G0G1 40.26
G2M 28.60
S 31.14
G0G1 48.61
G2M 19.53
S 31.85
G0G1 50.17
G2M 18.6
S 31.23
G0G1 39.42
G2M 29.79
S 30.79
Number
Channel(PE-A)
Cell cycle distribution(%)
G0/G1
S
G2/M
C
P27
PLAGL2
cyclin D1
CDK4
cyclin E
Relative mRNA level
MMP9
Ncadherin
Vimentin
Ecadherin
D
HE
E-cadherin
N-cadherin
Vimentin
Cyclin D1
CDK4
Cyclin E
E
PLAGL2 Score
E-cadherin Score
N-cadherin Score
Vimentin Score
CDK4 Score
Cyclin D1 Score
Cyclin E Score

Figure S2

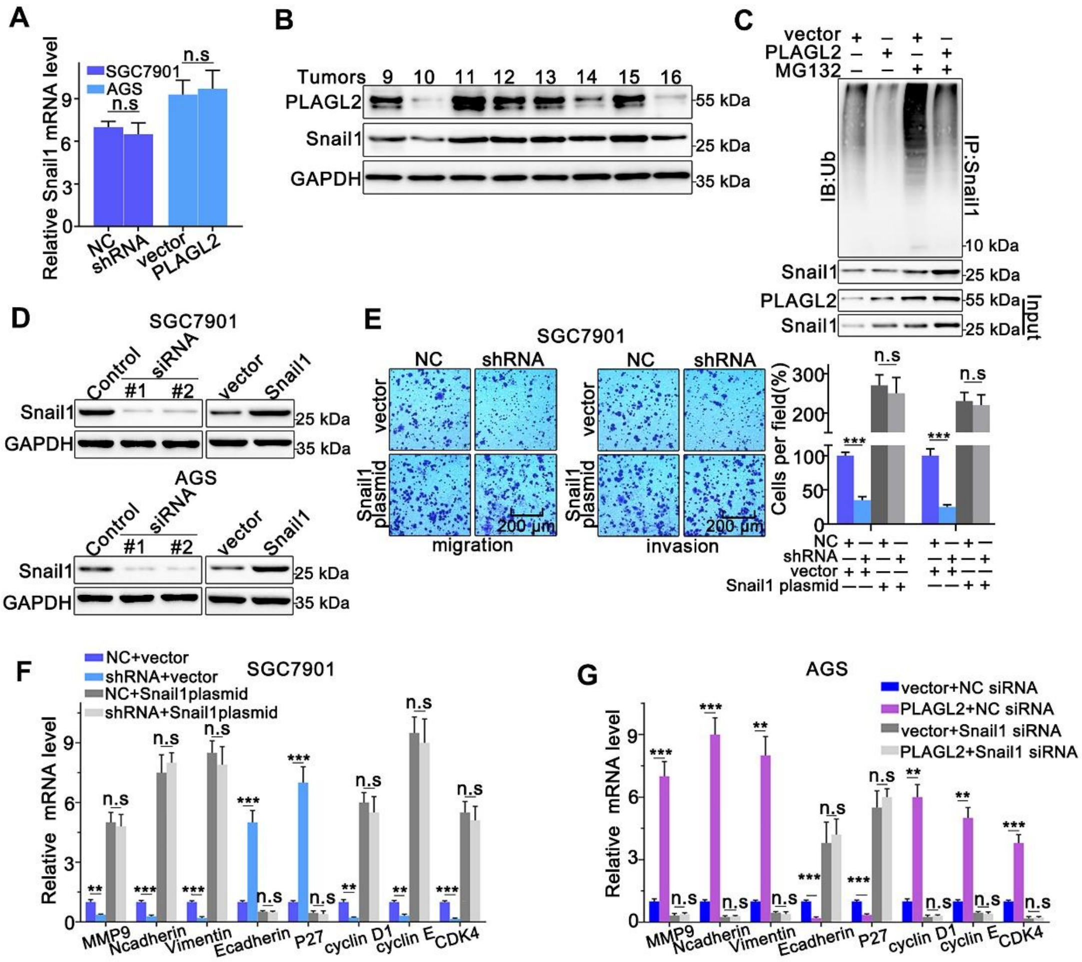
A
Relative Snail1 mRNA level
SGC7901
AGS
n.s
NC
shRNA
vector
PLAGL2
B
Tumors 9 10 11 12 13 14 15 16
PLAGL2
Snail1
GAPDH
55 kDa
25 kDa
35 kDa
C
vector
PLAGL2
MG132
IB:Ub
IP:Snail1
10 kDa
Snail1
PLAGL2
Input
D
SGC7901
AGS
Control
siRNA
#1
#2
vector
Snail1
GAPDH
E
NC
shRNA
Snail1 plasmid
200 μm
migration
invasion
Cells per field(%)
F
NC+vector
shRNA+vector
NC+Snail1plasmid
shRNA+Snail1plasmid
Relative mRNA level
MMP9
Ncadherin
Vimentin
Ecadherin
P27
cyclin D1
cyclin E
CDK4
G
vector+NC siRNA
PLAGL2+NC siRNA
vector+Snail1 siRNA
PLAGL2+Snail1 siRNA

Figure S3

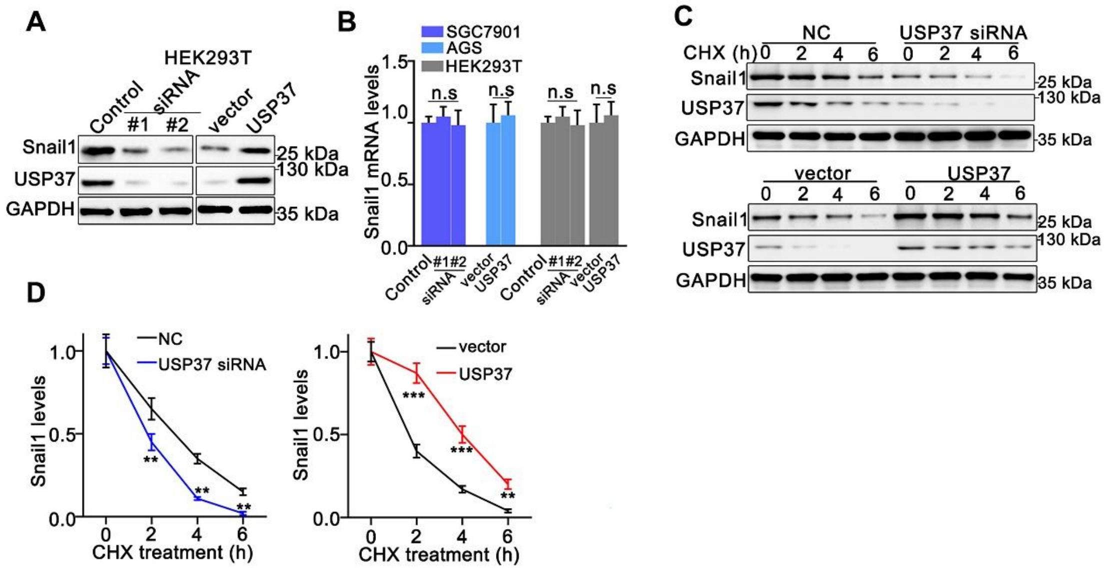
A
HEK293T
Control
siRNA
#1
#2
vector
USP37
Snail1
USP37
GAPDH
25 kDa
130 kDa
35 kDa
B
SGC7901
AGS
HEK293T
Snail1 mRNA levels
1.5
1.0
0.5
0.0
n.s
Control
#1#2
siRNA
vector
USP37
C
NC
USP37 siRNA
CHX (h)
0
2
4
6
vector
Snail1
USP37
GAPDH
25 kDa
130 kDa
35 kDa
D
NC
USP37 siRNA
vector
USP37
Snail1 levels
CHX treatment (h)
**
***

Figure S4

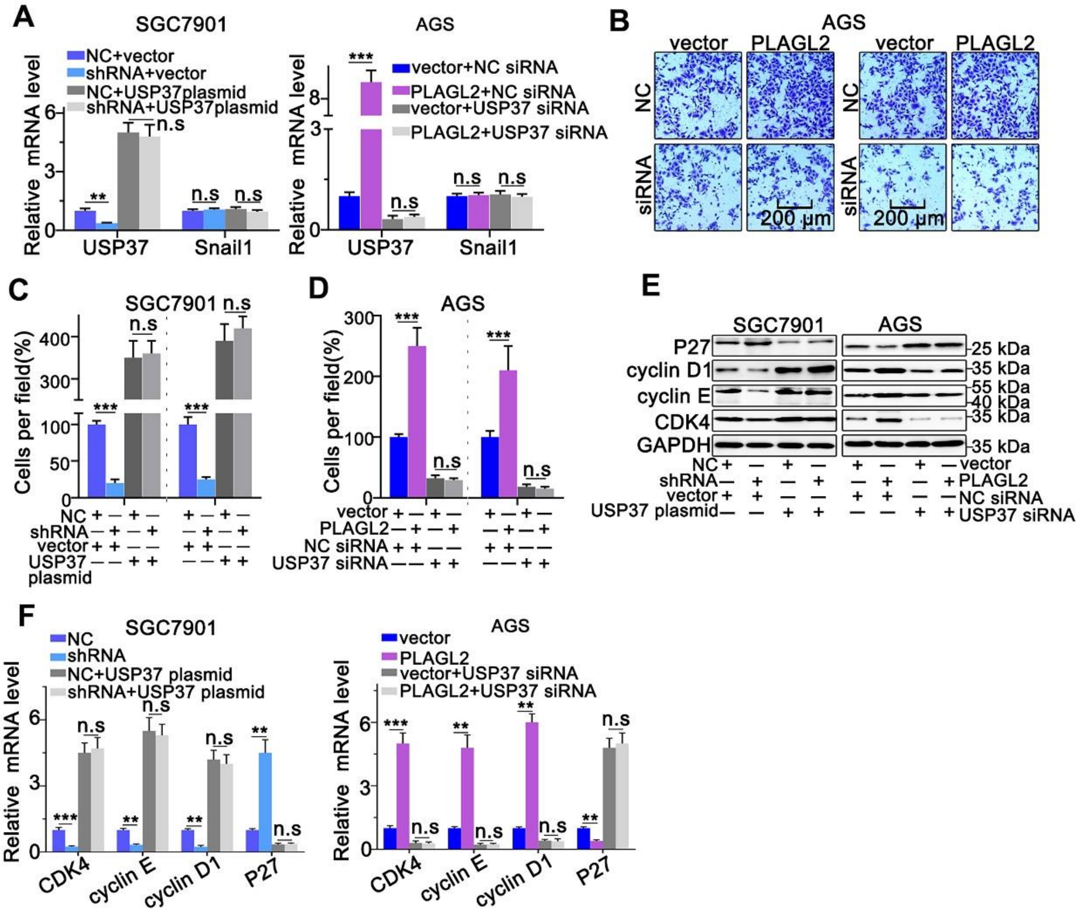
A
SGC7901
NC+vector
shRNA+vector
NC+USP37plasmid
shRNA+USP37plasmid
Relative mRNA level
USP37
Snail1
n.s
AGS
vector+NC siRNA
PLAGL2+NC siRNA
vector+USP37 siRNA
PLAGL2+USP37 siRNA
B
AGS
vector
PLAGL2
NC
siRNA
200 μm
C
SGC7901
Cells per field(%)
NC
shRNA
vector
USP37 plasmid
D
AGS
vector
PLAGL2
NC siRNA
USP37 siRNA
E
SGC7901
AGS
P27
cyclin D1
cyclin E
CDK4
GAPDH
25 kDa
35 kDa
55 kDa
40 kDa
35 kDa
35 kDa
NC
shRNA
vector
USP37 plasmid
vector
PLAGL2
NC siRNA
USP37 siRNA
F
SGC7901
NC
shRNA
NC+USP37 plasmid
shRNA+USP37 plasmid
Relative mRNA level
CDK4
cyclin E
cyclin D1
P27
AGS
vector
PLAGL2
vector+USP37 siRNA
PLAGL2+USP37 siRNA
